# Measurements of the trapezius and erector spinae muscles using virtual touch imaging quantification ultrasound-Elastography: a cross section study

**DOI:** 10.1186/s12891-017-1733-8

**Published:** 2017-08-25

**Authors:** Anne Heizelmann, Sümeyra Tasdemir, Julian Schmidberger, Tilmann Gräter, Wolfgang Kratzer, Beate Grüner

**Affiliations:** 1 Department of Internal Medicine I, Ulm University Hospital, Albert-Einstein-Allee, 23 89081 Ulm, Germany; 2 Department of Diagnostic and Interventional Radiology, Ulm University Hospital, Albert-Einstein-Allee 23, Ulm, 89081 Germany; 3 Department of Internal Medicine III, Ulm University Hospital, Albert-Einstein-Allee 23, Ulm, 89081 Germany

**Keywords:** Virtual touch imaging quantification, Muscles, Ultrasound Elastography, Trapezius, Erector spinae

## Abstract

**Background:**

This study uses virtual touch imaging quantification (VTIQ) technology for the first time to conduct measurements of the trapezius and erector spinae muscles in a large study population. The significance of various influencing factors, such as age and sex, are also examined.

**Method:**

The study population comprised 278 subjects. The Siemens Acuson S3000 and VTIQ technology were used for measurements of the trapezius and erector spinae muscles (Siemens Healthcare, Erlangen, Germany).

**Results:**

The following mean values ± standard deviation were calculated: left trapezius: males 2.89 ± 0.38 m/s, females 2.71 ± 0.37 m/s; right trapezius: males 2.84 ± 0.41 m/s, females 2.70 ± 0.38 m/s; left erector spinae: males 2.97 ± 0.50 m/s, females 2.81 ± 0.57 m/s; right erector spinae: males 3.00 ± 0.52 m/s, females 2.77 ± 0.59 m/s. A significant difference between male and female subjects was demonstrated both for the shear wave velocities of the trapezius and erector spinae as well as for the thickness of the trapezius muscle (*p* < 0.05). There was also a significant difference in muscle elasticity between subjects over 60 years of age and those under 60 (*p* < 0.05). Furthermore, the results indicate that regular physical activity has an effect on muscle elasticity.

**Conclusions:**

Our results demonstrate significantly different results between male and female subjects and between under- and over-sixty-year-old subjects. This means that sex-related and age-adapted considerations are obviously needed for further studies.

## Background

Tissue elasticity can be affected by pathological processes such as inflammation or tumours and is of diagnostic significance for many diseases [1]. The elasticity of muscle tissue is also subject to change due to various diseases, resulting for example in fatty depositions or fibrosis [2]. An increase in collagen fibres is also found, for example, in cases of sarcopoenia [3–6]. Several studies have shown that the elasticity of muscle tissue is affected both in congenital and acquired muscle diseases [6–10].

The possibility of measuring and quantifying these changes in the elasticity of muscle tissue can provide further information about the pathogenesis and pathophysiology of muscular disorders and can be used in a clinical setting for establishing a diagnosis, following up on the disease course and monitoring therapy [6, 11, 12].

Ultrasound elastography was developed in 1991 by Ophir et al. and represents a method for determining and measuring changes in tissue elasticity more exactly [13]. Virtual touch imaging quantification technology is a form of shear wave elastography and was used in this study to measure shear wave propagation velocity in tissue. Tissue elasticity can be determined based on the fact that shear wave velocity increases with progressive stiffness of the tissue [6, 11, 14].

We examined the trapezius and erector spinae muscles in this study because muscle pain and stiffness commonly present in the back and neck regions [12]. Myofascial trigger points are often found in the trapezius, but the present state of research in this field has not entirely understood their pathophysiology and pathogenesis. Further research is also made difficult by the lack of objective examination methods, as currently there is no other recognised diagnostic criterion apart from simple palpation [7, 11, 12, 15, 16]. Shear wave elastography represents a further possible examination method in addition to B-mode ultrasound imaging, in which trigger points are displayed by their reduced echogenicity. Shear wave elastography is able to measure objectively the mechanical properties of trigger points, which appear harder in comparison with non-affected muscle [7, 8, 12, 16]. The role of the muscles of the back (such as, for example, the erector spinae) and of the fasciae in the development of pain in the region of the lower back is the subject of an increasing number of research projects [17], since low back pain is very common with a life-time prevalence of 75–84% [18]. Possible examination methods for assessing lumbar muscles include, for example, magnetic resonance imaging and computed tomography [19]. Thus, a number of studies were able to show that increased fat content in the erector spinae or multifidus is associated with pain in the lower part of the back [20–23]. At present, there are no studies available which have examined the erector spinae muscle of patients with low back pain using shear wave elastography, and the question arises as to whether pathological alterations in the erector spinae of patients with low back pain can also be detected by shear wave elastography.

The aim of this study was to conduct measurements of the trapezius and erector spinae muscles using virtual touch imaging quantification technology in a large study population to obtain reference ranges which can be used as a basis for further studies. Furthermore, these values were to be assessed for the effect of any influencing factors such as sex, age, BMI, physical activity and right- or left-handedness.

## Methods

### Study design

The study was conducted between March 2013 and August 2013 in the Ultrasound Department of Ulm University Hospital. Exclusion criteria included previous surgery, fractures and injuries of the spinal region or shoulder joint area, intervertebral disc prolapse within the last ten years, use of anabolic agents, pregnancy, diabetes mellitus, osteoporosis, rheumatic disorders, inadequate depiction of the trapezius and erector spinae muscles on B-mode ultrasound imaging, and poor quality of the elasticity measurements. The entire study population comprised 324 subjects, 46 of which were excluded because of the mentioned exclusion criteria. The final study population therefore comprised 278 subjects between the ages of 18 and 73 years (110 males and 168 females). The average BMI of the subjects was 22.94 ± 2.81 and the average age was 35.51 ± 14.93 years.

### Ultrasound device and measuring method

The Siemens Acuson S3000 (Siemens Healthcare, Erlangen, Germany) and virtual touch imaging quantification (VTIQ) technology were used for this study, allowing a quantitative and qualitative assessment of tissue elasticity in one single measurement.

### Study design

The subjects were given a subject information sheet, an informed consent form and a questionnaire which asked them to give relevant information about themselves, such as age, sex, BMI, previous illnesses, operations, injuries, right- or left-handedness, and the extent of physical activity. In addition they were also asked about frequent backache and headache. Examination of the back muscles was conducted with the subjects positioned prone with their arms at the side of their body. The examination couch had a recess at the top end so that the subjects did not need to turn their head to one side and could lie in a relaxed manner. The subjects had to lie resting for three minutes on the examination couch before the measurements were started to allow the muscles to be as relaxed as possible at the time of measuring. Ten different examiners familiar with the use of B-mode ultrasound imaging examined the subjects.

### Erector spinae muscle

**Fig. 1:**
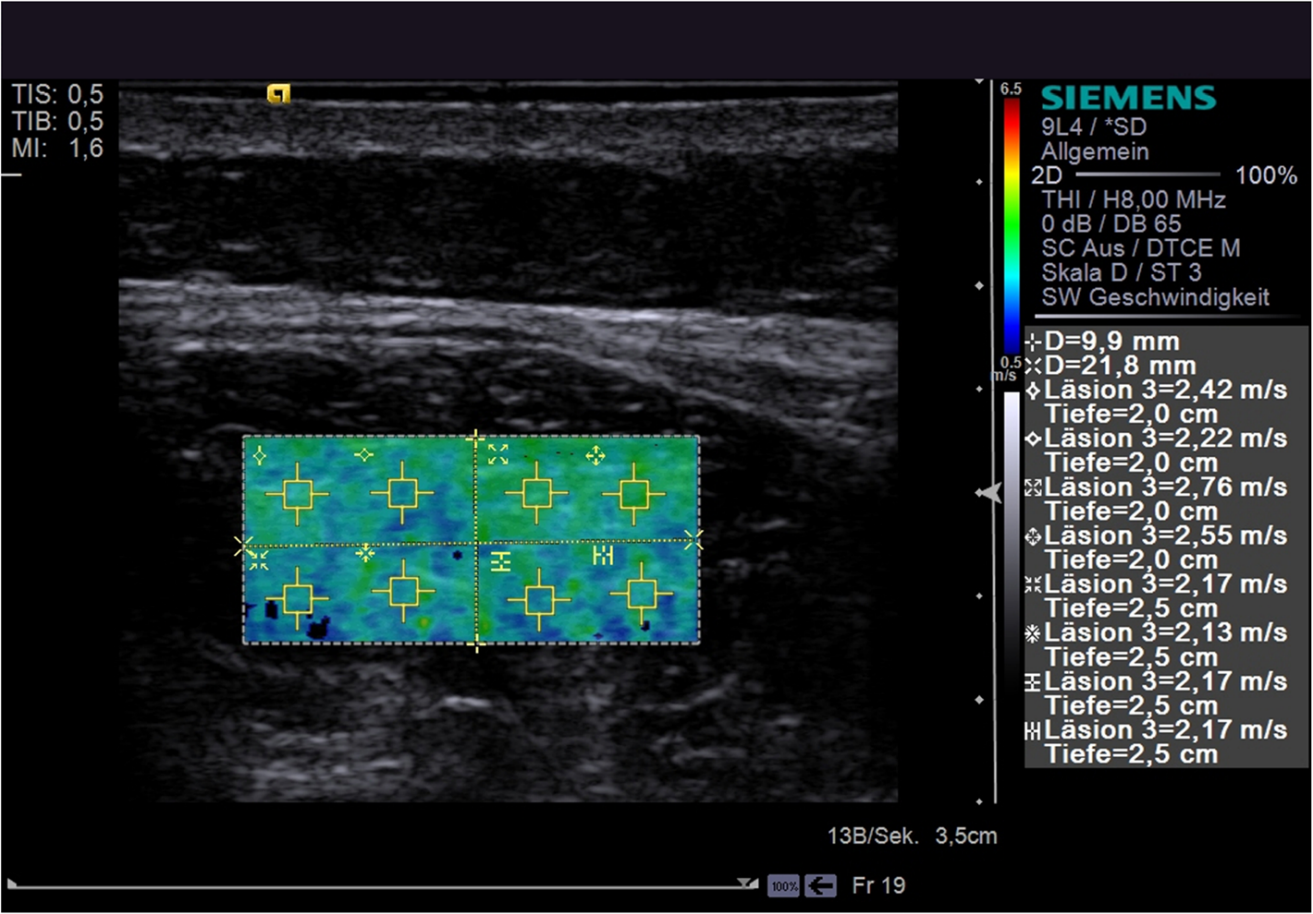
Examination of the erector spinae muscle. Qualitative and quantitative measurements of the shear wave velocities using virtual touch tissue imaging quantification (Acuson S3000, Siemens) of the erector spinae muscle (shear wave speeds ranging from 0.5 m/s – 6.5 m/s: *blue* = low shear wave velocities, *green* = moderate shear wave velocities, *red* = high shear wave velocities) (Ulm University Hospital, 2013)

To examine the erector spinae muscle, the transducer was placed transversely immediately adjacent to the spine, on the right and left side, at the level of the iliac crest, without applying pressure to the skin. The measurements were made successively, each along the lateral tract of the erector spinae muscle. Firstly, a B-mode image was obtained, after which the ROI box for the subsequent elasticity measurement was placed approx. 5 mm below the superficial lamina of the thoracolumbalis fascia. Next, the VTIQ measurements were carried out: a colour-coded elastogram (blue = soft tissue; red = hard tissue) was created and eight ROIs were placed within the previously defined box for the quantitative measurement of the propagation velocities of the shear waves. The ROI box was divided into four quadrants, with two ROIs placed in each quadrant (Fig. 1).

### Trapezius muscle

To examine the trapezius muscle, the transducer was placed transversely from a dorsal approach on the right and left side at the level of the vertebra prominens and the mid-clavicular line. The thickness of the right and left trapezius was measured on the B-mode image and the transducer was aligned longitudinally to the muscle fibres within the transverse part of the trapezius muscle. The ROI box was adjusted on the right and left, depending on the thickness of the trapezius, and positioned. Subsequently, the VTIQ measurements were performed, for which purpose eight ROIs were positioned, each on the right and left side, with again two ROIs per box quadrant.

### Statistical evaluation

The statistical analysis system (SAS) Version 9.2 of the North American SAS Institutes, Cary, North Carolina, was used for calculating absolute and relative frequencies and for calculating t-tests (normally distributed) and Wilcoxon two-sample tests (not normally distributed). The Shapiro-Wilk test was used to test the data for normal distribution. A significance level of alpha = 0.05 was chosen. The graphs were created using the table calculation program Microsoft Excel 2013 by Microsoft Corporation, Redmond, USA, based on the statistics obtained from the SAS.

## Results

### Mean values and 95% confidence intervals

The mean values ± standard deviation and 95% confidence interval were calculated for the shear wave velocities of the trapezius and erector spinae muscles and for the thickness of the trapezius. Calculating the 95% confidence interval provides a reference range for the present study (Table 1).

**Table 1:** Mean values ± standard deviation and 95% confidence intervals

		Total (*n* = 278)	Males (*n* = 110)	Females (*n* = 168)
Left trapezius [m/s]	Mean ± SD	2.78 ± 0.38	2.89 ± 0.38	2.71 ± 0.37
	95% confidence interval	[2.73–2.83]	[2.81–2.96]	[2.65–2.77]
Right trapezius [m/s]	Mean ± SD	2.75 ± 0.37	2.84 ± 0.41	2.70 ± 0.38
	95% confidence interval	[2.71–2.80]	[2.76–2.92]	[2.64–2.76]
Left erector spinae [m/s]	Mean ± SD	2.87 ± 0.55	2.97 ± 0.50	2.81 ± 0.57
	95% confidence interval	[2.80–2.93]	[2.87–3.06]	[2.72–2.89]
Right erector spinae [m/s]	Mean ± SD	2.86 ± 0.57	3.00 ± 0.52	2.77 ± 0.59
	95% confidence interval	[2.80–2.93]	[2.90–3.10]	[2.68–2.86]
Thickness left trapezius [mm]	Mean ± SD	11.94 ± 3.05	13.47 ± 3.07	10.93 ± 2.59
	95% confidence interval	[11.58–12.30]	[12.89–14.05]	[10.54–11.32]
Thickness right trapezius [mm]	Mean ± SD	11.95 ± 2.88	13.21 ± 3.03	11.13 ± 2.44
	95% confidence interval	[11.61–12.29]	[12.64–13.79]	[10.75–11.50]

### Sex

**Fig. 2:**
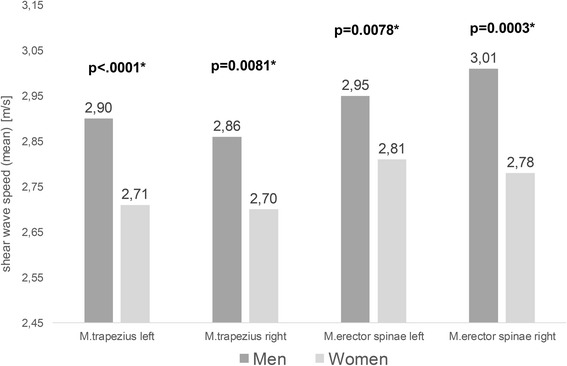
Shear wave velocities in males and females. Difference between the shear wave velocities of the trapezius and erector spinae muscles in male subjects (*n* = 110) and female subjects (*n* = 168); * = statistically significant (Ulm University Hospital, 2013)

There was a significant difference between male (*n* = 110) and female subjects (*n* = 168). Male subjects had significantly higher values for shear wave velocity for the trapezius and erector spinae muscles as well as for the thickness of the trapezius muscle (*p* < 0.0001) (Fig. 2).

### Age

The study population was divided into one group with subjects ≤60 years of age (*n* = 254) and a second group with subjects >60 years (*n* = 24 (17 ♂, 7♀)). There was a significant difference between these two groups regarding shear wave velocities of the right and left erector spinae muscles and of the right trapezius; the shear wave velocities were higher in the over-sixty-year-olds (Table 2).

**Table 2:** Shear wave velocities in subjects aged ≤60 and >60 years

*N* = 278	Mean ± SD ≤ 60 years	Mean ± SD > 60 years	z-value	*p*-value
Left trapezius	2.78 ± 0.38	2.81 ± 0.38	0.3953	0.3463
Right trapezius	2.74 ± 0.41	2.91 ± 0.23	2.05613	0.0052*
Left erector spinae	2.85 ± 0.52	3.11 ± 0.75	1.7048	0.0441*
Right erector spinae	2.84 ± 0.58	3.07 ± 0.49	1.9641	0.0248*

Data not normally distributed: Wilcoxon Two-Sample Test (z-values); * = significant

### Physical activity of the participants

**Fig. 3:**
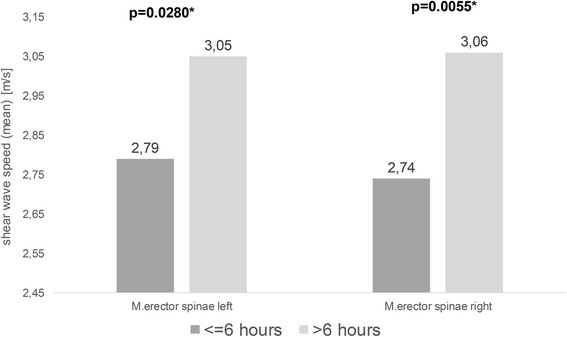
Physical activity. Difference in shear wave velocity of the erector spinae muscle, depending on the physical activity of the 18- to 30-year-old subjects, specified in hours per week (subdivided into ≤ 6 h per week (*n* = 131) and >6 h per week (*n* = 24); * = significant (Ulm University Hospital, 2013)

A subgroup was formed comprising subjects aged 18–30 years (*n* = 155). Physical activity, specified in hours per week, correlated with the shear wave velocities of the erector spinae muscle (left: *p* = 0.0021, right: *p* = 0.0187). No significant correlation was evident in the trapezius muscle (left: *p* = 0.2024, right: *p* = 0.3902). Physical activity of the subjects was subdivided into ≤6 h per week (*n* = 131) and >6 h (*n* = 24). A significant difference in the thickness of the trapezius was demonstrated on comparing the two groups: (left: *p* = 0.0249, right: *p* = 0.0230) and in the shear wave velocities of the erector spinae muscle (left: *p* = 0.0280 and right: *p* = 0.0055) (Fig. 3). No significant difference was evident in the trapezius muscle (left: *p* = 0.6497, right: *p* = 0.3272).

### Correlation between the thickness of the trapezius and muscle elasticity

The thickness of the trapezius muscle correlated with the of the erector spinae. A slight correlation was found in the trapezius muscle only between the thickness of the right trapezius and the shear wave speeds of the right trapezius (Table 3). Calculation of the correlation between shear wave velocities and BMI did not produce any unequivocal results. A significantly negative correlation was evident between the shear wave velocities of the erector spinae and BMI (*p* < 0.05). There were no significant correlations in the comparison of healthy subjects and subjects with frequent backache (*n* = 65), headache (*n* = 56) or scoliosis (*n* = 44). Nor were there any significant results in the comparison between right hand-dominant (*n* = 242) and left hand-dominant individuals (*n* = 22). The calculation of differences in shear wave velocities of the trapazius and spinae regarding adipose people (BMI > 25 and <25) adjusted for right and left handed probands showed no significant results and differences (*p* > 0.05).

**Table 3:** Correlation between the thickness of the trapezius and muscle elasticity

*N* = 278		Left trapezius	Right trapezius	Left erector spinae	Right erector spinae
Thickness left trapezius	Correlation coefficient r	0.10468	0.11260	0.25657	0.18751
	*p*-value	0.0826	0.0617	<.0001*	0.0018*
Thickness right trapezius	Correlation coefficient r	0.04784	0.14225	0.26859	0.16656
	*p*-value	0.4286	0.0180*	<.0001*	0.0055*

* = significant

## Discussion

According to our present state of knowledge, this is the first study to perform measurements of the trapezius and erector spinae muscles using virtual touch imaging quantification (VTIQ) technology and the Siemens Acuson S3000 (Siemens Healthcare, Erlangen, Germany). A significant advantage of this study is its large patient population consisting of 278 subjects, as there are currently no studies available with a comparably large study population. Using the Acuson S2000 and virtual touch tissue imaging quantification (VTTQ) technology, Kuo et al. calculated a mean value of 2.0865 ± 0.4480 m/s with their measurements of the trapezius muscle of 20 subjects, a value that is lower than the mean values calculated by the present study for the trapezius [24]. Since Kuo et al. examined their subjects seated, and therefore in a different position from that used in the present study, and also used different Siemens software applications, a comparison between the two studies does not provide very meaningful information.

There are differences between the muscles of males and females, with females, for example, having less muscle strength, a smaller muscle cross-section area and a slower muscle contraction compared to males [4]. With their measurements of the masseter and gastrocnemius muscles of 127 subjects (Supersonic Shear Imaging), Arda et al. demonstrated significantly higher shear wave velocities for males compared to females [1]. Akagi et al. also reached this result with their measurement of the rectus femoris, gastrocnemius and soleus muscles of 80 subjects (Acuson S2000, Siemens Healthcare, Erlangen, Germany) [3]. Akagi et al. assume that the difference in muscle thickness of males and females is the reason for the gender-specific differences in shear wave velocities found in several studies [3]. Kuo et al. examined the trapezius, anterior scalene, levator scapulae and sternocleidomastoid muscles and found no significant difference between males and females (Acuson S2000, Siemens Healthcare Erlangen) [24]. However, the study population was comparatively small with 20 subjects, nine of which were male and 11 female, which rather restricted the informative value of this study. The present study demonstrated that male subjects had significantly higher shear wave velocities in their trapezius and erector spinae muscles and a significantly thicker trapezius compared to female subjects. This result supports the assumption of Akagi et al. that the higher shear wave velocities in the muscle tissue of males are related to the greater muscle thickness compared to females [3]. Since the majority of the studies demonstrated a significant difference in muscle elasticity and/or stiffness, these results are important with regard to use of shear wave elastography in patients with musculoskeletal symptoms and disorders and show that a sex-specific differentiation is necessary when determining reference values.

The ageing process of muscles already sets in at about the age of 25, although this does not result in any significant morphological changes until about the sixth decade of life. Only after about the sixth decade does this process start to accelerate, the number, quality and strength of the muscle fibres decrease, while collagen content on the other hand increases [3, 4]. According to our state of knowledge, these alterations of the muscles with increasing age, known as sarcopoenia, result in increased stiffness of the muscle [3, 4]. After creating two age groups (≤ 60 and >60 years), the present study revealed a significant difference between these two entities with regard to the right and left erector spinae muscles and the right trapezius. No significant difference was demonstrated for the left trapezius, which could be due to the fact that the sub-group of over-sixty-year-olds only comprised 24 subjects, while the group of under-sixty-year-olds consisted of 254. Further studies with approximately the same number of subjects in each group would be helpful in confirming this age-related difference in muscle elasticity. Eby et al. confirmed the results of the present study with their measurements of the biceps brachii muscle in 133 subjects between the ages of 21 and 94 years (Supersonic Shear Imaging) in as much as they also demonstrated higher shear wave velocities in subjects over 60 years of age [4]. Akagi et al., on the other hand, did not discover any greater shear wave velocities in the over-sixty-year-olds [3]. Since Akagi et al. examined muscles of the lower limbs, while the present study and that of Eby et al. measured muscles of the upper limbs and back muscles, it is difficult to make any comparison between the studies and to attempt to explain their discrepancies [3]. It may be worth considering in this respect whether muscles of the lower limbs are more affected by the living circumstances and everyday activities of older people than the muscles of the upper limbs. Furthermore, the issue should be clarified as to whether during the process of sarcopoenia the above alterations affect and alter the properties of muscles of the upper and lower limbs to the same degree.

The results regarding the relationship between shear wave velocities and physical activity show that regular sport does have an effect on stiffness and elasticity of the erector spinae muscle and on the thickness of the trapezius. It should be noted that for the trapezius no significant difference and no significant correlation of the shear wave velocities were evident in relation to physical activity, although the thickness of the trapezius muscle was significantly influenced by a physical activity of more than six hours sport per week. In this regard, it is noticeable that on calculating the correlation between the thickness of the trapezius muscle and the shear wave velocities of the trapezius and erector spinae, significant correlations were evident between the thickness of the trapezius muscle and the shear wave velocities of the erector spinae muscle. It should also be noted that the thickness of the trapezius muscle did not correlate with the shear wave velocities of the trapezius. There is therefore an association between the shear wave velocities of the erector spinae and the thickness of the trapezius, which is related to the results of calculations of the dependency of shear wave velocities on physical activity. There are practically no studies available which have examined the relation between physical activity and muscle elasticity in a similar fashion. Eby et al. also enquired about the degree of physical activity in their 133 subjects, but found no significant connection between the shear wave velocity of the biceps brachii and the physical activity of the subjects [4]. Therefore, the results of the present study indicate that increased regular physical activity changes the elasticity of muscle tissue. This relationship should be examined more exactly and be reassessed in further studies. It would also be important to take into account the type and intensity of the various physical activities, e.g. whether they mainly involve strength or endurance and which muscle groups are being trained.

The results of the measurements did not reveal any significant difference between subjects with backache and headache and those without these symptoms. Although the questionnaire did ask about frequent headache and backache, this was a yes/no question, and the subjects were not asked about the quality, duration, location and frequency of occurrence of the symptoms. Since the results of the subjects with backache and headache did not show any significant difference from those without symptoms, these subjects remained in the study population. The fact that headache and backache are very common symptoms only serves to support this approach. An aim of further studies would be to compare the values obtained in healthy subjects with those of patients with specifically ascertained, examined and located muscle-related backache and headache. In their study using the VTTQ technique, Kuo et al. examined the relationship between neck pain and shear wave velocities of the trapezius muscle. They asked their subjects about neck pain which had lasted longer than six months. They found a significant difference in shear wave velocities between subjects with and without pain involving the trapezius muscle; however, only three of 20 subjects reported neck pain [24]. The small number of participants and the very low number of subjects with symptoms did, however, somewhat restrict the informative value of this study.

## Conclusions

The essential results regarding the clinical use of shear wave elastography in muscle tissue are the significantly different values obtained between male and female subjects and between subjects under and over the age of 60, making it clear that normal value ranges need to be adapted to sex and age.

These results obtained in the present study provide a basis for further studies examining the relation between muscle pain/stiffness and the shear wave velocity of muscle tissue or, following on, the significance of various influencing factors for muscle tissue and thus reassessing the clinical application of shear wave elastography in muscles.

## Abbreviations

BMI: Body-Mass-Index
m/s: Meters per second
Mean: Average
mm: Milimeter
ROI: region of interest
SAS: Statistical Analysis System
SD: standard deviation
VTIQ: virtual touch imaging quantification
VTTQ: virtual touch tissue imaging quantification
